# Analytical Characterization and Inhibitor Detection in Liquid Phases Obtained After Steam Refining of Corn Stover and Maize Silage

**DOI:** 10.3389/fchem.2021.760657

**Published:** 2021-10-14

**Authors:** Malte Jörn Krafft, Jens Berger, Bodo Saake

**Affiliations:** Chemical Wood Technology, University of Hamburg, Barsbüttel, Germany

**Keywords:** corn stover, maize silage, steam refining, carbohydrates, inhibitors, by-products

## Abstract

The utilization of agricultural products and residues for the production of value-added and biobased products is a highly relevant topic in present research. Due to the natural recalcitrance of lignocellulosic biomass against enzymatic degradation, pretreatments are important requirement for further processes. For the raw material in this study, corn stover (CS) as highly available agricultural residue and maize silage (MS) as model substrate for an ensiled agricultural product were pretreated by steam refining. However, after processing a liquid fraction and fibers are present. Subsequent to steaming the fiber fraction is well characterized. Nonetheless, in depth characterizations of the filtrates are also important for their subsequent utilization. Decreasing molar masses from 7,900 g/mol to 1,100 g/mol for CS filtrates and 100.000–12.900 g/mol for MS filtrates were determined with increasing severity. Due to their proven inhibitory effect on microorganisms weak acids, furans and phenolic compounds within the liquid phased were analyzed. Especially formic acid increases with increasing severity from 0.27 to 1.20% based on raw material for CS and from 0.07 to 0.23% based on raw material for MS. Further GC/MS measurements indicate, that up to 8.25% (CS filtrate) and 5.23% (MS filtrates) of the total peak area is related to inhibitory phenols. Considering the data, detoxification strategies are of non-negligible importance for filtrates after steam refining and should be considered for further research and process or parameter optimizations. An alternative may be the application of milder process conditions in order to prevent the formation of inhibitory degradation products or the dilution of the gained filtrates.

## Introduction

Maize (*Zea mays* L.) is one of the most abundant crops in the world. Beside the utilization as food and the usage for industrial materials it furth is a well-known model organism for several fields of research (Strable and Scanlon, 2009). The high availability of maize and its products are displayed in estimates for the worldwide corn production. Based on data of the Food and Agriculture Organization of the United Nations (FAO) and literature values for the straw/kernel-ratio, Krafft et al. (2020b) calculated an estimated annual maize production of 2.15–2.64 billion tons for 2018. Updated FAO data for 2019 show quite similar values (FAOSTAT, 2020).

However, the usage of lignocelluloses and lignocellulosic residues in biorefinery contexts is highly discussed and due to the aforementioned high availability, maize is focused on research. Therefore, two types of utilization based on maize can be distinguished: the usage of the whole plant and the usage of components, like kernels, straw or cob waste. Therefore, the usage of the whole plant, mostly after ensiling to extend the storage stability for the production of biogas is described. Further, the usage of the kernels is reported to produce e.g., bioethanol (Lebuhn et al., 2008; Schwietzke et al., 2009). However, the usage of these two parts is associated with debates about land use (Graebig et al., 2010) and the production of coarse grains for energy instead of primary food grains (Srinivasan, 2009).

Due to these debates, the usage of agricultural post-harvest residues is beneficial, although there are debates about sustainable collection with consideration of soil erosion and water conservation (Cruse and Herndl, 2009). With that in mind, corn stover (CS) is characterized as an undervalued harvest residue after grain threshing, which remains mainly on the field and decomposes (Glassner et al., 1998). Nonetheless, lignocellulosic biomass is recalcitrant against natural biodegradation. Therefore, a pretreatment of the used CS is necessary to reduce this known recalcitrance (Himmel et al., 2007).

Seasonal availability and the storage of lignocellulosic biomass is a key factor for a full-year biomass supply of a biorefinery plant. These aspects were discussed in previous studies (Cherubini and Strømman, 2011; Miao et al., 2012; Giuliano et al., 2016). In that context ensiling was mentioned in literature as a suitable storage method for biomass in biorefineries (Oleskowicz-Popiel et al., 2011). Moreover, maize silage (MS) has a lower grade of recalcitrance against enzymatic degradation, mainly due to lower lignin contents, but a pretreatment is also necessary to receive sufficient results after enzymatic hydrolysis (EH) (Krafft et al., 2020b). In the last years steam refining was conducted for pretreatments of wood (Schütt et al., 2011; Schütt et al., 2012), forest residues (Janzon et al., 2014), CS (Krafft et al., 2020a), MS (Krafft et al., 2020b) or waste medium density fiberboards (MDF) (Hagel and Saake, 2020). Most studies were conducted to enhance the enzymatic digestibility for the production of monomeric carbohydrates, but attempts for MDF fiber recycling after steam refining are reported as well (Hagel et al., 2021).

Like mentioned before, a biomass or raw material pretreatment is a necessity for a lignocellulose biorefinery. Beside different pretreatment methods, the most widely described pretreatment is steam explosion, working as a physicochemical process. Due to the knowledge, that the explosion step is unnecessary for the enhancement of EH (Brownell et al., 1986), several authors are currently discussing an alternative steam pretreatment where the defibration is conducted by a refining step. Therefore, this variant is also called steam refining (Schütt et al., 2011; Schütt et al., 2012; Janzon et al., 2014). The process results in two fractions, one being a solid fiber fraction, the other being a hydrolysate. However, in literature on steam pretreatment different terms are used for the liquid: extract phase or fraction, liquid phase or fraction, solubilized or (water)-soluble phase or fraction or (pre)-hydrolysate and filtrate. All of the above equally paraphrase the identical fraction, a clear nomenclature is yet no present. Therefore the term “filtrate” will consequently be used throughout this publication. While the usage of the cellulose-rich fiber fraction is often addressed in literature, the utilization of the hydrolysate has been less frequently investigated (Cubas-Cano et al., 2020). A plausible explanation would be the challenging fermentation of C5 sugars with wild-type microorganisms and the abundance of inhibitors in the hydrolysates (Cubas-Cano et al., 2018; Cubas-Cano et al., 2020).

The formation and liberation of degradation products from carbohydrates and lignin and their inhibitory effect on enzymes and bacteria is well documented (Cantarella et al., 2004). Cantarella et al. (2004) differs three groups of toxic compounds: low molecular weight acids, carbohydrate degradation products and lignin degradation products. In this context Zhang et al. (2013) compared eleven potential inhibitors based on previous studies of Palmqvist and Hahn-Hägerdal (2000) and Klinke et al. (2004). From early studies of Ando et al. (1986) it is known that most of the degradation products are soluble within the water-phase and only a few aromatic monomers are detectable within the solid phase after steaming. Nonetheless, Tengborg et al. (2001) report on the inhibiting effect on EH and fermentation. Different amounts of prehydrolysates were added to the solid phase followed by EH. Adding prehydrolysates lowered the cellulose conversion rate continuously. Further, the authors investigated the influence of washing the solid fraction and came to the conclusion, that washing is beneficial for fermentation processes (Tengborg et al., 2001). Qureshi et al. (2010) investigated the fermentation of CS hydrolysates to acetone butanol ethanol (ABE) after pretreatment. No microbial growth or fermentation products were measured in the undiluted and untreated liquid phase. Fermentation started 40 h after dilution and addition of sterile glucose. Further treatments, like dilution with other hydrolysates and overliming with Ca(OH)_2_ were necessary for sufficient fermentation. The authors suggest the investigation of processes without inhibitory by-product formation or the development of inhibitor resistant cultures for fermentation. In the past, several studies accounting the inhibitory content of the hydrolysates were conducted for gardening residues (Cubas-Cano et al., 2020), trimming vine shoots (Bustos et al., 2005), softwood (Shi et al., 2015), wheat straw (Aulitto et al., 2017), sugarcane bagasse (van der Pol et al., 2016) or oil palm empty fruit bunches (Ye et al., 2014). Inhibitors’ influences on the hydrolysate after steaming is to be considered proven due to these studies. It furthermore is significant to include this issue in contemplations of pretreatment conditions.

Former studies depict yields, characterization and utilization of the fiber fraction after steam refining (Krafft et al., 2020a; Krafft et al., 2020b). In this study, a detailed analytical characterization of filtrates obtained after steam refining of CS (Krafft et al., 2020a) and MS (Krafft et al., 2020b) will be described to complete a full overview about the obtained fractions. Therefore, results from carbohydrate analysis and molar mass distribution, as well as results on organic acids and furans will be presented. Additionally, information on inhibitory compounds obtained by GC-MS are going to be given. All data is evaluated under consideration of the used severity factor while steaming. All in all, the goal was to gain a more detailed overview over the received filtrates after steam refining for further processes, like whole-slurry processes or simultaneous EH and fermentation to value-added products.

## Materials and Methods

### Raw Material

The filtrates were collected from previous steam refining studies with 200 g raw material input based on CS and MS (Bendler, 2018; Krafft et al., 2020a; Krafft et al., 2020b; Frey, 2020). Briefly, steam refining is content-wise related to steam explosion with the difference, that the defibration is conducted by a mechanical defibration instead of a pressure relief. All results are recalculated to weight percent based on the CS and MS raw material and are denoted as % RM.

Steam refining was conducted from 160°C up to 200°C for 10 min, respectively. For a better comparability with available literature the severity factor according to Overend and Chornet (1987), expressed as log = R_0_, was calculated based on Eq. 1. A list of temperatures, duration of exposure and corresponding severity can be gathered from Table 1.

$$\log R_{0}=\log\;\left(t\;\times \;e^{\frac{\left(T-100\right)}{14.75}}\right)$$ (1)

**TABLE 1:** List of pretreatment conditions and corresponding severity factors.

Temperature [°C]	Time [min]	Severity factor
160	10	2.77
170	10	3.06
180	10	3.36
190	10	3.65
200	10	3.94

The hydrolysate was collected by filtration through a sieve and was afterwards freeze-dried (Alpha 2-4 LSC; Martin Christ Gefriertrocknungsanlagen GmbH, Osterode am Harz, Germany). A detailed description of the process is given in the aforementioned studies. For reasons of completeness, the carbohydrate and lignin composition of the used lyophilizates will be shown in Table 2.

**TABLE 2:** Composition of the used lyophilizates in % based on the filtrate fraction.

	Log R_0_	Gluc	Xyl	Arab	Others^a^	Residue
CS	2.77	12.2	3.7	2.7	3.7	9.1
	3.06	10.9	7.3	3.6	3.5	11.9
	3.36	8.7	13.9	4.7	3.5	11.8
	3.65	5.4	23.1	3.0	3.0	9.5
	3.94	5.2	24.2	2.4	3.0	1.5
MS	2.77	59.1	3.3	2.0	0.5	2.2
	3.06	55.7	6.4	2.2	0.7	3.1
	3.36	51.5	9.6	2.2	1.2	5.5
	3.65	52.5	9.6	1.8	1.1	5.8
	3.94	47.3	8.9	1.4	1.1	7.2

a Containing Rhamnose, Galactose, and Mannose.

### Acidic Hydrolysis

The hydrolysis of the freeze-dried steam refining filtrates was conducted as one-step acidic hydrolysis according to Lorenz et al. (2016). Therefore, 100 mg of the lyophilizate were added to 10 ml demineralized water and were homogenized in an ultra-sonic water bath. Afterwards, 1.8 ml H_2_SO_4_ was added and the samples were hydrolyzed in an autoclave for 40 min at 120°C and 0.12 MPa. After autoclaving the samples were cooled down to room temperature, filtered through G4 sintered glass frites and were collected for borate-HPAEC carbohydrate analysis. The residue was collected in the sintered glass frit and was determined gravimetrically after drying at 105°C ± 3°C over night.

### Chromatographic Methods

For the detection of carbohydrates borate-HPAEC (Dionex™ UltiMate™ 3,000, Thermo Fisher Scientific™, Waltham, MA, United States) was used with an anion exchange resin (MCI GEL® CA08F, Mitsubishi Chemical, Tokyo, Japan) according to an in-house protocol. Two potassium tetraborate/boric acid buffers (pH 8.6 and 9.5) were used in different concentrations. Post-column derivatization was conducted at 65°C and carbohydrate detection was performed at a wavelength of 560 nm. For more details of this method see also Lorenz et al. (2016). Oligomers were detected as difference between total free carbohydrates in the filtrates and total carbohydrate content after two-step acidic hydrolysis.

Furans, here 5-HMF and furfural, were determined by RP-HPLC directly after steaming in order to avoid secondary degradation reactions. Therefore, 20 µL of post steaming hydrolysate was separated at 25°C for 80 min using an AQUASIL™ C_18_ column (250 × 4.6 mm × 5 μm; Thermo Fisher Scientific™, Waltham, MA, United States). Weak acidic water and acetonitrile were used as eluents in different concentrations with a flow rate of 1 ml/min^−1^. For a more detailed description of the used method see Krafft et al. (2020a).

Ion chromatography for organic acid detection was performed using a Dionex™ ICS 2000 with an IonPac^TM^ AS11-HC (2 × 250 mm) anion exchange column (Dionex, Sunnyvale, CA, United States). Potassium hydroxide (KOH) reaching from 1 to 70 mM was used as eluent with a flow rate of 0.38 ml/min^−1^ at 35°C.

Size exclusion chromatography was performed according to a reported method for xylans by Saake et al. (2001). 3 GRAM columns (8 × 300 mm, Polymer Standards Service) with 30, 1,000 and 3,000 Å pore size were used with a mixture of DMSO and Water (9:1) and 0.05 mol Lithium bromide. Measurements were conducted at 60°C and a flow rate of 0.4 ml/min^−1^ using a refractive index detector (RI-71, Shodex). For molar mass calibration pullulan standards (Polymer Standards Service) were used.

Sample preparation for GC/MS analysis was conducted as thermally assisted hydrolysis and methylation (THM) using tetramethylammonium hydroxide (TMAH) according to Becerra and Odermatt (2013). Therefore, 120 µg of the sample were prepared with 10 µL TMAH (10% in water) and was dried for 12 h above P_2_O_5_.

THM-GC/MS was performed using a Frontier Lab Micro furnace Multi-shot pyrolyzer (EGA/Py-3030iD) combined with an autosampler (AS-1020 E). The pyrolysis system was interfaced to a GC/MS (6,890/5973N Agilent Technologies, United States). The temperature for thermochemolysis was set at 350°C, interface temperature at 330°C, the inlet and the GC/MS interface temperature were kept at 320°C. A low polarity ZB-5HT column (30 m × 0.25 mm i. d., 0.25 µm film thickness) was used with helium as carrier gas. The split ratio was set to 30:1. A flow rate of 1 ml/min was set for gas chromatographic separation. Oven program starts with 4 min at 45°C, changes to 5°C/min up to 340°C and kept 15 min at 340°C. Mass spectra was determined by a 5973 N agilent inert MS with 70 eV electron impact ionization energy. Scan range was 15–550 m/z. Evaluation was performed using an in-house and NIST 20 database. Therefore, substances were detected as methyl derivatives.

## Results and Discussion

### Size Exclusion Chromatography (SEC)

**FIGURE 1:**
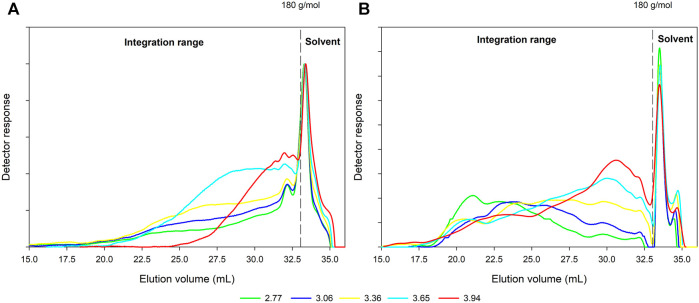
SEC elution profiles of filtrates obtained from CS **(A)** and MS **(B)**.

SEC was applied in order to investigate the influence of increasing steaming severity on the molar mass and molar mass distribution of components dissolved in the filtrate of CS and MS. In that context, Figure 1 and Table 3 depict the molar mass distribution of CS and MS filtrates.

**TABLE 3:** Average molar mass (M_w_) in g/mol and dispersity (Đ) measured by SEC for CS and MS filtrates.

Log R_0_	CS					MS				
	2.77	3.06	3.36	3.65	3.94	2.77	3.06	3.36	3.65	3.94
M_w_	7,900	7,500	6,850	4,900	1,100	100.000	60.400	59.600	44.000	12.900
Đ	13.4	10.3	8.5	5.6	2.1	20.1	20.5	28.1	30.6	13.4

The intensity and shape of the molar mass distributions for the CS filtrate (Figure 1A) indicated, that an increase of severity from log R_0_ = 2.77 to 3.65 result into a continuous increase of the peak area correlating with an increased amount of dissolved components. The weight average molar mass (M_w_) is continuously reduced from 7,900 g/mol to 4,900 g/mol (Table 3). The molar mass distribution curves are very broad with a dispersity of 13.4 for the lowest severity (Figure 1A, Table 3). The dispersity decreases with increasing severity parallel to the M_w_ reduction. At the highest severity of log Ro 3.94 an increased degradation of extracted components is apparent. The high molar mass shoulder in the elution profiles disappears while low molar mass components are more prominent. This shift results in a low molar mass of 1,100 g/mol and a low dispersity of 2.1.

A more complex picture emerges for the molar mass distribution of MS filtrates (Figure 1B). At the lowest severity of log R_0_ = 2.77, a pronounced high molar mass peak is visible around 21 ml. Krafft et al. (2020b) reported a starch content of 38.6% for the used raw material. It can be assumed, that due to the solubility of starch, high amounts of starch or rather big starch fragments are present in the hydrolysate. This high molar mass fraction in the rather broad distribution curve results in a high M_w_ of 100.000 g/mol and show a high dispersity of 20.1 (Tab. 3) as well. This starch is degrading successively with increasing severity and the peak is shifting to higher elution volumes.

Again, with higher severity an increased peak area is indicating a higher proportion of dissolved components. At highest severity, the highest proportion is attributed to low molar mass components eluting between 30–32.5 ml again. However, in contrast to CS, MS filtrates still contain high molar mass components, even at the highest severity. Therefore, even at log R_0_ = 3.94 the M_w_ and the dispersity remain high with 12.900 g/mol and 13.4, respectively (Tab. 3).

In earlier works, Puls et al. (1985) investigated the influence of an increasing severity on the so called “hemicellulose fraction” of birch. The authors also report a shift to lower molar masses with an increase of the severity. Similar results were reported e.g. by Montané et al. (1998) after steam explosion of wheat straw. The authors performed SEC for washing water, which is similar to the filtrate in this study. They also report a strong decrease of the molar mass with severity, starting with an M_w_ of around 11.000 at a severity of log R_0_ = 3.39 and resulting in an M_w_ of 400 at log R_0_ = 4.13. Montané et al. (1998) describe the molar mass decrease with depolymerization reactions of compounds with higher molar mass to oligomers and monomers. The hydrolytic cleavage of the glycosidic bonds of cellulose and hemicelluloses during steaming to monomers and the further degradation to e. g., 5-HMF and furfural is therefore described in the literature (Li et al., 2005). As main reaction of the lignin, also β-O-4′ structures are depolymerized during steaming and the solubilized lignin occurs in the filtrate (Montané et al., 1998; Li et al., 2007).

In summary, a decrease of M_w_ with increasing severity is visible for both samples. Nonetheless, filtrates from MS consistently show much higher molar masses compared to CS. The primary driver for that finding is most likely starch from the kernels in the silage fraction.

### Monomer and Oligomer/Polymer Detection

**FIGURE 2:**
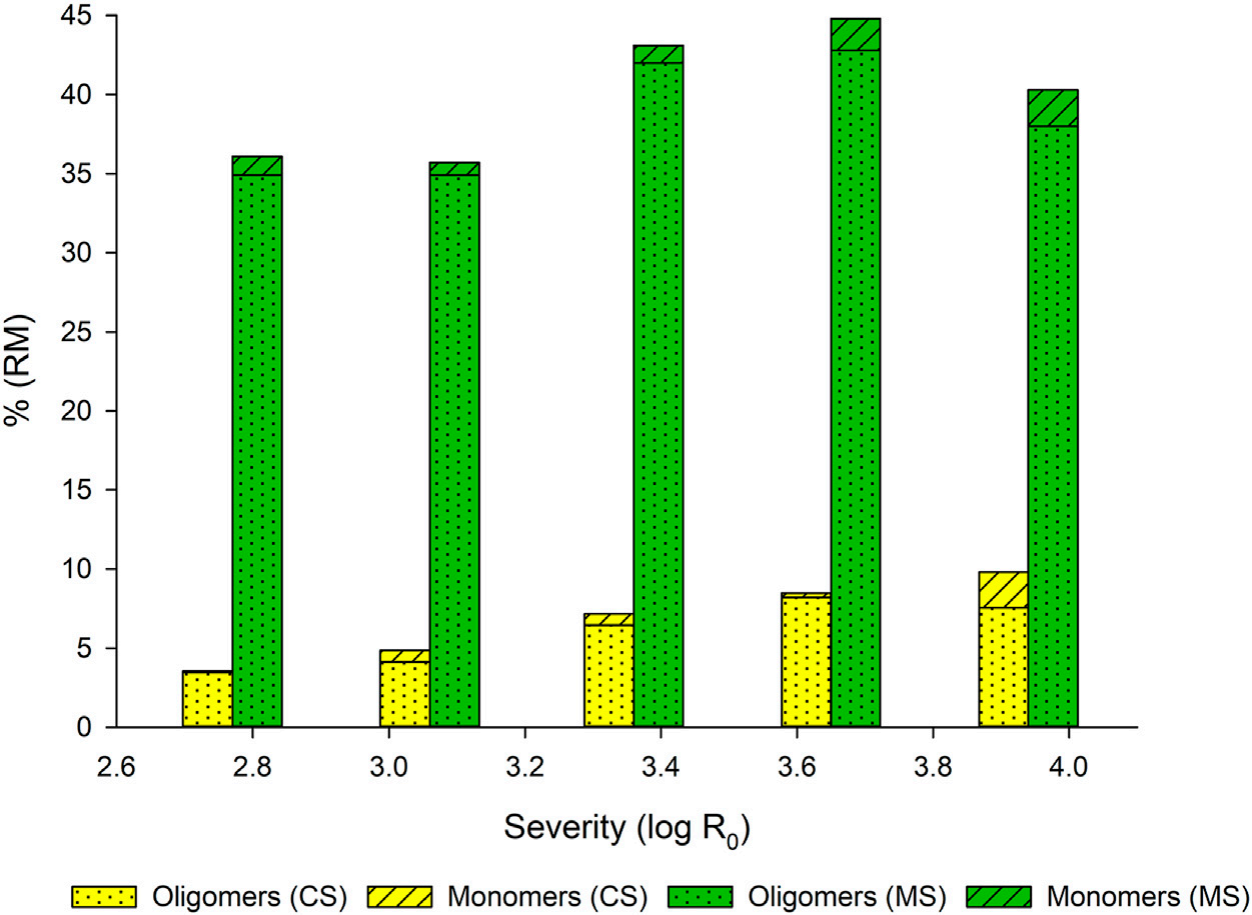
Effect of steaming severity on monomer and oligomer/polymer ratio in filtrates obtained from CS and MS.

The detection of free monomeric carbohydrates in the filtrates after steaming was performed to calculate the oligomer/polymer fraction. Therefore, the known carbohydrate content after acidic hydrolysis was compared with the detected monomers prior to hydrolysis. The difference between these two values represents the oligomeric/polymeric carbohydrate fraction. Oligomers, especially xylooligomers, are also reported as inhibitory compounds for cellulases (Kumar and Wyman, 2009). For this reason, oligomers are mentioned in this section (Figure 2).

Different developments are described in Figure 2. High carbohydrate contents, as mentioned before originating from soluble starch, were determined in the MS filtrate. The relative oligomer/polymer proportion varies between 97.8% (log R_0_ = 3.06) and 94.3% (log R_0_ = 3.94). The highest absolute monosaccharide content of 2.3% was detected in filtrates treated at the highest severity. However, at this severity, also the total carbohydrates in the filtrate are reduced, indicating intensified degradation reactions at high pretreatment conditions. In contrast, CS filtrates show much lower total carbohydrate contents. Low monomer values are detected up to a severity of log R_0_ = 3.65, with a relative oligomer/polymer proportion approximately at 90%. At the highest severity (log R_0_ = 3.94), the absolute monomer content is equal to reported for MS filtrates. At this severity, the oligomer/polymer proportion is around 77% of the total carbohydrates.

Several authors mention the changes of mono- and oligomer ratios during steaming. Known mechanisms for polymer degradation to oligomers/monomers are hydrolytic processes, leading to a cleavage of the glycosidic bonds of the polymers, e. g. cellulose and hemicelluloses (Li et al., 2005). Romero-García et al. (2016) investigated olive leaves pretreated between 180 and 220°C. The authors reported the highest carbohydrate recovery for pretreatment conditions of 180°C for 10 min. At this point, 64% of the released carbohydrates originated in the oligomeric form. The authors found oligomer contents above 60% for all examined conditions. Sharma et al. (2015) compared the influence of sulfuric acid impregnation on the oligomer release from rice straw. After a pretreatment at 200°C for 10 min (log R_0_ = 3.94), they found 60% of the pentoses as oligomers and 75% of the hexoses, respectively. After impregnation with sulfuric acid and pretreatment at 180°C for 10 min, much lower values of oligomers (20.5% pentoses, 40.4% hexoses) were determined. The authors concluded that catalyzed steaming conditions are beneficial in order to avoid inhibition by oligomers for subsequent processes. Nunes and Pourquie (1996) compared steam explosion of *Eucalyptus globulus* (Labill.) wood chips with and without acid catalysts. They concluded that higher severities are needed under non-acidic conditions for the same carbohydrate release from the substrate and efficient following EH. Furthermore, they reported, that an efficient oligomer hydrolysis was not detected under non-acidic conditions. For steam explosion experiments of sunflower stalks without acid catalysts Ruiz et al. (2008) report an average of 90% oligomers in the filtrates. These data is quite similar to the present study. The authors postulate for such high oligomer contents the necessity of a so-called “posthydrolysis” to make the oligomers accessible for fermentation approaches. These considerations for filtrates with high oligomer/polymer values can be adopted for the filtrates investigated in the present study.

### Detection of Inhibitors

#### Weak Organic Acids

**FIGURE 3:**
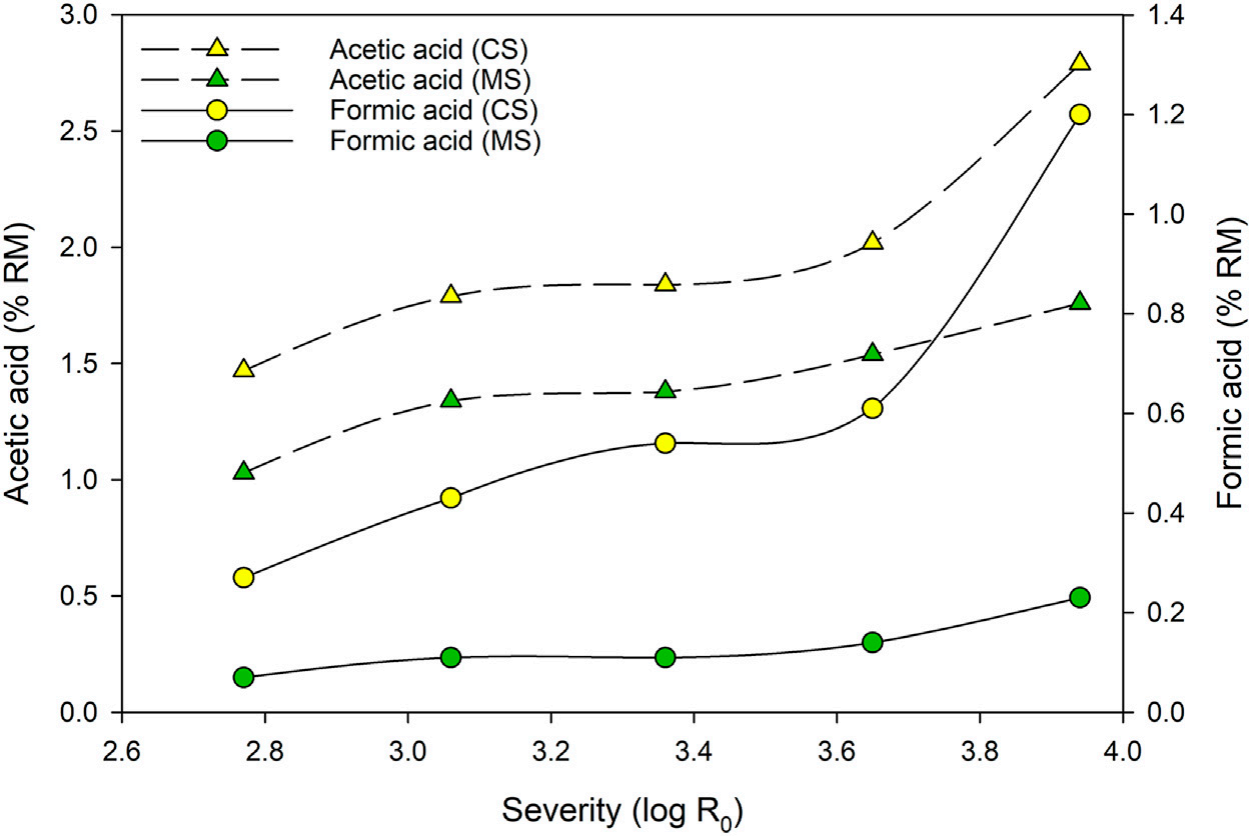
Effect of steaming severity on the acetic and formic acid content of CS and MS filtrates.

Organic acids and furans are often mentioned as the main inhibitor fractions with amounts of 82–96% of all inhibitors (Zhang et al., 2013). However, acetic and formic acid were identified as the main organic acids in the process. Acetic acid originates therefore from hemicellulose-related acetyl groups. Due to autoionization of water between 150 and 230°C, hydronium ions are formed. These ions work as catalysts and conduct a cleavage of the hemicellulose bonded acetyl groups. Further, hydronium ions originating from acetic acid also act as catalysts for the degradation of polysaccharides. Formic acid is formed by degradation of furfural and 5-HMF, originating from pentose and hexose degradation (Pedersen and Meyer, 2010; Rasmussen et al., 2014). Since the dilution of the hydrolysate differs after the steaming, all results were calculated as % RM. Figure 3 shows acetic and formic acid calculations for both, MS and CS filtrates.

An increase of formic and acetic acid, as main aliphatic carboxylic acids, appears with increasing severity. Acetic acid, originating from acetyl groups of xylan hemicelluloses in the used CS, is increasing from 1.47% RM up to 2.79% RM. For MS, the liberated acetic acid concentration is lower and increases from 1.03% RM up to 1.76% RM. Similar developments occur for formic acid, although at lower levels between 0.27–1.20% RM for CS and 0.07–0.23% RM for MS. The increase of organic acids with an increase of the applied severity is also reported on in the area of expertise’s literature. Hagel and Saake (2020) report similar trends for steam refining between log R_0_ = 2.47 and log R_0_ = 3.95 of poplar and spruce wood chips. They report acetic acid contents of 0.18–1.83% RM for poplar and 0.04–0.7% RM for spruce, respectively. Further they report formic acid contents between 0.02–0.37% RM (poplar) and 0.02%–0.26 (spruce). Whereas the trends are similar, the acid contents are lower compared to the present study. Steam explosion experiments with sunflower stalks between log R_0_ = 3.05 and 4.53, reported by Ruiz et al. (2008), show strong increases, mainly between severities of log R_0_ = 3.64 and 4.23. However, whereas the acetic acid are in an equal range, the formic acid concentrations of Ruiz et al. (2008) are significantly higher compare to this study. Summarizing, the trends found in this study and in available literature, an increased formation of weak acids for higher severities is generally reported. However, the absolute amount of weak acids formed depends on the used substrate.

Regarding the inhibitory effect of the organic acids on enzymatic hydrolysis and subsequent fermentation to ethanol, Cantarella et al. (2004) report, that acetic acid concentrations of 2 g/L did not affect the activity of enzymes. Feng et al. (2012) also found no inactivation using an acetic acid concentration of 2 g/L and Oliva et al. (2003) tested 10 g/L without reduction of the ethanol production. Captured in Figure 3, more than 2.5% RM (CS) and more than 1.5% RM (MS) acetic acid were found at the highest severity and a dilution of the obtained filtrates may be necessary. However, formic acid shows a strong influence for a lactic acid fermentation at concentrations of 0.1 g/L using bush clovers (*Lespedeza crytobotrya* Miq.) stalks (Feng et al., 2012). In the present data, around 1.2% RM (CS) formic acid were detected. However, less containing formic acid were determined for the MS samples. A dilution or detoxification of the filtrates, mainly from CS, is therefore needed prior to fermentation.

Due to the known mechanism of hydronium ion formation, process conditions below 150°C may be beneficial to prevent autoionization and acetic acid formation in steam refining processes. Nonetheless, Krafft et al. (2020b) tested steam refining below 150°C for MS and found nearly stable values for acetic acid. Therefore, free lactic acid in the ensiled sample may be responsible for degradation reactions and the cleavage of hemicellulose-bonded acetyl groups. Therefore, lactic acid is usually not formed during steaming. Nonetheless, it is known as inhibitor formed during fermentation processes (Cubas-Cano et al., 2020). It is important to know that the filtrates obtained from MS contain relevant amounts of lactic acid originating from the raw material. Krafft et al. (2020b) point out lactic acid contents for the used filtrates between 5.87% RM (log R_0_ = 1.59) and 4.79% RM (log R_0_ = 4.12) with a slight decrease for higher severities. Due to the knowledge of lactic acid naturally occurring in MS, lowering organic acid fractions in the process conditions being beneficial to the CS process, but superfluous towards the MS process, seems like a plausible hypothesis.

#### Furan Derivatives

Further degradation products of the carbohydrates are the furan derivatives 5-hydroxymethylfurfural (5-HMF) and furfural. 5-HMF is known as one of the most important molecules obtained from biomass and is reported to be formed under acidic conditions from the dehydration of mainly monosaccharides. Further reactions, like the formation to levulinic and formic acid or the polymerization of 5-HMF and intermediates to humins are reported (Souza et al., 2012). Further, Danon et al. (2014) report mechanisms and kinetics of furfural formation from pentoses by dehydration. However, the authors show multiple options for the formation of furfural deriving from pentose.

Values for furan contents in the filtrates after steam refining of CS and MS were reported by Krafft et al. (2020a) and Krafft et al. (2020b). Due to the known instability, furans were detected directly after steaming in the filtrates. Further a recalculation to the biomass input was performed for better comparability. Fufural and 5-HMF are known inhibitors. From experiments with the filtrate of poplar, steam exploded at 210°C for 4 min, Oliva et al. (2003) report, that furfural is twice as toxic than 5-HMF. However, experiments indicate the relevance of these products. They will therefore be briefly compared and discussed for the sake of completeness.

**FIGURE 4:**
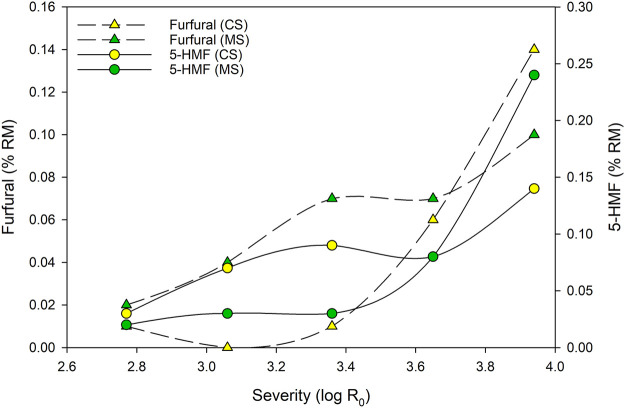
Effect of steaming severity on the concentration of furan derivatives (furfural and 5-HMF) in the filtrates obtained from CS and MS.

As illustrated in Figure 4, increasing numerical values for 5-HMF and furfural are detected. Hagel and Saake (2020) show similar developments for the mentioned furan derivatives after steam refining at different severities of poplar and spruce. Therefore, furfural contents are quite similar and at the highest severity (log R_0_ = 3.94) around 0.15% RM furfural is reported for both, poplar and spruce. Much lower values for 5-HMF are reported by the same authors, resulting in approximately 0.05% RM 5-HMF at a severity of log R_0_ = 3.94.

From the known mechanism of 5-HMF and furfural formation under acidic conditions, steaming under neutral process conditions may be a suitable way for furan derivative reduction. Hagel and Saake (2020) report steam refining of waste MDF between pH 7 to 8 with nearly no measured furfural and 5-HMF production. Due to known degradation reactions provoked by low or high pH values, neutral conditions may be a way for process optimization to prevent both, furan derivative formation and cellulose/hemicellulose degradation.

### Phenolic Compounds

**FIGURE 5:**
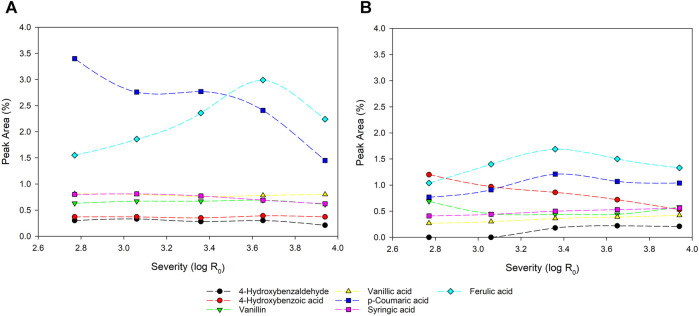
Peak area % in GC/MS of the main phenolic compounds for CS **(A)** and MS **(B)**. The peak area is calculated as percentage based on the area of the whole chromatogram.

As mentioned before, phenolic degradation products and fragments are determined in the filtrates originating from the plant lignin. In Figures 5A,B the relative peak areas of the relevant inhibitory phenolics in GC/MS data after TMAH derivatization are depicted.

Origins and formation mechanisms of the measured compounds are commonly reported on in various publications. Nonetheless, in regards to lignified biomass and maize silage, containing kernels, multiple origins especially for ferulic acid are possible. For lignified substrate, ferulic acid is linked covalent to lignin and originates from the oxidative attack of coniferyl alcohol (Mathew and Abraham, 2006). Ferulate esters are reported as major linkages in grasses with the formation of “lignin-ferulate-polysaccharide”-complexes (LFP) (Ralph et al., 1995). Ferulic acid occurs also in maize kernels and therefore, the kernels in the maize silage are a further origin of ferulic acid (Sen et al., 1991). The conversion of ferulic acid to vanillin is reported with six different pathways for conversion (Mathew and Abraham, 2006). Vanillin is further reported as depolymerization product of lignin, obtained e.g., by oxidation, alkaline processes or severe temperature/pressure conditions. However, a confirmed reaction mechanism is reported controversial since many parameters influence the reaction sequence (Fache et al., 2016). Also, the cleavage of β-O-4′ bonds is reported to be a mechanism for the depolymerization of lignin to vanillin and vanillic acid (Rinesch et al., 2017). *p*-coumaric acid is reported to be a naturally present hydroxycinnamic acid in the cell walls of wood like ferulic acid as well (Lam et al., 2001). The formation of 4-Hydroxybenzoic acid is reported by Rasmussen et al. (2016). The authors show a cleavage of lignin side chains and the formation of 4-Hydroxybenzoic acid after hydrothermal treatment. It is to be mentioned, that follow-up sequences between other degradation products are possible. (Rasmussen et al., 2016). Closing, 4-Hydroxybenzaldehyde is described as aldehyde monomer of lignin (Hu et al., 2018) and syringic acid is reported as small phenolic degradation fragments from S and G-type lignins (Longe et al., 2020).

As stated, ferulic acid and *p*-coumaric acid represent the main phenolic molecules detectable in the filtrate. These fractions show the most significant change in concentrations with increasing severity. For CS, a high proportion of *p*-coumaric acid is found at low severity which decreases constantly at harsher reaction conditions. This indicates that *p*-coumaric acid is easily released from the lignin but undergoes secondary reactions, like degradation or reactions with other degradation products, at harsher conditions. Ilanidis et al. (2021) investigated hydrothermal pretreatment of wheat straw and found a reduction of *p*-coumaric acid at temperatures higher than 190°C. In the present study, ferulic acid is also increasing up to a severity of log R_0_ = 3.65 and is then decreasing at the point of the highest severity. For MS both compounds show an increase up to log R_0_ = 3.36 followed by a reduction at the highest severity. However, in general these compounds were detected in much lower levels compared to corn stover filtrates. In that context Gong et al. (2012) also report degradation and polymerization of phenolic compounds at higher severities. Early works of Fiddler et al. (1967) report 4-methylguaiacol, 4-ethylguaiacol, 4-vinylguaiacol and vanillin as thermal degradation products of ferulic acid.

Further lignin degradation products, like 4-hydroxybenzaldehyd, 4-hydroxybenzoic acid, vanillin, vanillic and syringic acid are nearly stable for CS independent regarding the severity. These compounds were found in low amounts below 1% peak area. For the MS filtrates after steam refining, a more complex picture was obtained. A constant decrease with severity increase is visible for 4-hydroxybenzoic acid indicating secondary reactions. Syringic and vanillic acid are slightly increasing with the increasing severity. A noticeable increase of 4-hydroxybenzaldehyde is also reported by Ilanidis et al. (2021) for hydrothermal pretreatment of wheat straw. The authors suggest that phenolic degradation products with only one carbon side chain are not as sensitive for further degradation as compounds with longer side chains.

Zhang et al. (2013) also describe the influence of temperature and time on the behavior of the mentioned inhibitors. With increasing severity, they report increasing amounts of syringic acid and 4-hydroxybenzaldehyde, while stable figures were found for vanillin. Furthermore, a strong decrease with higher severities was reported for *p*-coumaric acid. Vanillic and 4-hydroxybenzoic acid were not found by Zhang et al. (2013).

**FIGURE 6:**
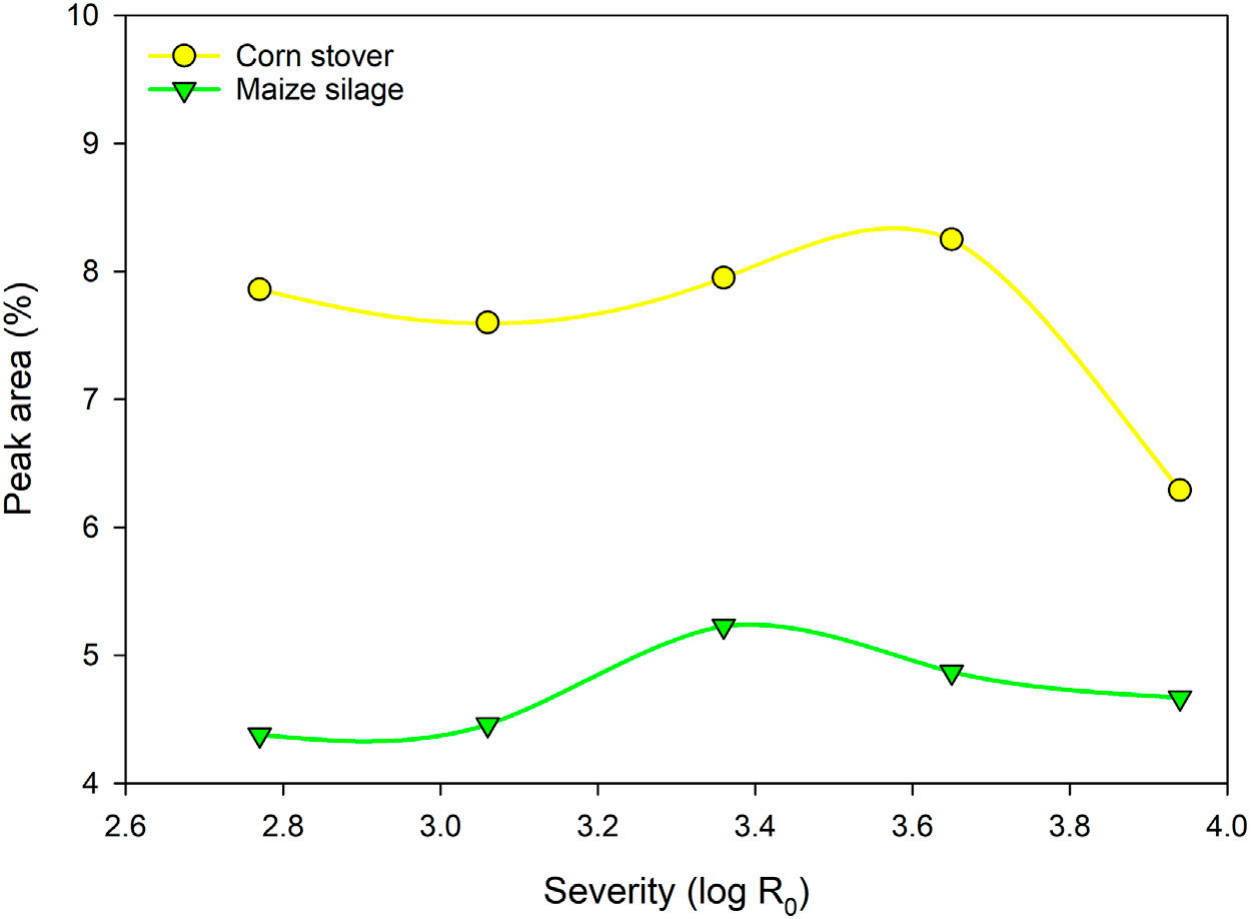
Summarized peak area % of the main phenolic compounds detected by GC/MS for CS and MS filtrates. The peak area is calculated as percentage based on the area of the whole chromatogram.

Comparing both substrates, much lower amounts of phenolic degradation products occur in MS filtrates (Figure 6). This is in accordance with the different lignin content of the two raw materials. Krafft et al. (2020a) report 19.3% lignin for the used CS, whereas 11.9% lignin are reported for the MS (Krafft et al., 2020b). With that in mind, higher proportion of phenolic degradation products could be expected for CS filtrates and it can be assumed, that the usage of substrates with low lignin contents is beneficial for the process. However, also a delignification of the substrate before steam refining is conceivable and is further known to enhance the enzymatic accessibility (Krafft et al., 2020a).

### Further Compounds Detected by GC

Beside the aforementioned inhibitors, further noteworthy compounds were detected. Propane-1,2,3-tricarboxylic acid was detected in MS samples most likely as breakdown product of a water soluble fumonisin. Fumonisins are known mycotoxins in maize, originating from a contamination with the mold fungus *Fusarium verticillioides* (Sacc.) Nirenberg (formerly *Fusarium moniliforme*), which is a common fungal pathogen in corn (Viquez et al., 1996; Munkvold and Desjardins, 1997; Marasas et al., 2004). Whereas the influence of such toxins is investigated for human beings or livestock, consuming contaminated maize, the possible influence for microorganism in biorefinery contexts is subject of recent research (Salati et al., 2014; De Gelder et al., 2018; Giorni et al., 2018) and up to now, depending on the fumonisin concentration, no or less negative influence of mycotoxins was found for biogas (Ferrara et al., 2021) and ethanol fermentation processes (Bothast et al., 1992).

Further compounds which also show an increase with increasing severity are e.g. 3,4-dihydrocoumarin-6-ol or alginetin. 3,4-dihydrocoumarin-6-ol is also reported by Oudia et al. (2007) in a Pyrolysis-GC/MS study of eucalyptus kraft pulps and by Kouhi and Shams (2019) after co-pyrolysis of bagasse and waste heavy paraffin. Alginetin is therefore reported as “caramelization product”, originating from pentoses and hexuronic acids after thermal treatments (Doi et al., 2020). However, both compounds are not mentioned in the context of inhibitory effects in the literature.

## Conclusion

Concluding this studies data, several inferences can be highlighted. Firstly, the obtained SEC data show a decrease of the molar mass of both samples with increasing severity. High molar masses around 100.000 g/mol were determined at the mildest condition for MS filtrates, with a reduction to 12.900 g/mol at the highest severity of log R_0_ = 4.12. Values for CS filtrates are much lower but a decrease of the determined molar mass with an increase of the severity is observed as well. The reason for the significant difference between MS and CS filtrates can be most likely attributed to starch from the kernels in the MS sample. The decrease in the molar mass is provoked by degradation reactions due to increasing severity and the resulting pH reduction induced by liberated organic acids in the filtrate.

The analysis of the ratio of monomers vs. oligomers/polymers in the obtained filtrates revealed that only for corn stover, treated at the highest severity, a significant amount (23% of the available carbohydrates) is obtained as monomers. All other conducted pretreatments lead to an oligomer/polymer content higher than 90%. Most of the carbohydrates are therefore not accessible for microorganisms. An EH prior to fermentation is a mandatory requirement for the obtained filtrates. However, processes like biogas production can be considered for the utilization of the filtrates, too.

Several observations were made for inhibitors in the filtrates. For the weak, organic acids mainly acetic and formic acid are in the focus for causing inhibitory effects. From the literature it is known that already concentrations of 0.1 g/L formic acid cause inhibitory reactions and a reduction of microbial activity. In the present study, relevant formic acid amounts were detected in the filtrates. Therefore, for the highest concentration (CS, log R_0_ = 3.94) a dilution is required to prevent effects, caused by the formic acid. Moreover, a detoxification or dilution of the obtained filtrates is necessary for further process steps in the context of a biorefinery concept. Considering the found furan derivatives and phenolic compounds, relevant amounts of both were found in the analyzed filtrates. Furfural increased up to 0.14% RM (CS) and 0.10% RM (MS). 5-HMF show an increase up to 0.14% RM (CS) and 0.24% RM (MS). Both compounds result from the degradation of pentoses and hexoses. A total amount of approximately 8% peak area can be put down to inhibitory, phenolic compounds for CS and 4–5% for MS, respectively. MS was proven to contain less phenolic compounds, mainly due to lower lignin contents in the raw material. However, the inhibitory activity on model organisms is subject to further research where steam refining filtrates must be tested in the context of EH and fermentation. Nonetheless, a direct fermentation is reported as insufficient, and a detoxification of similar filtrates was reported as a necessity within the literature.

Comparing both substrates, MS is beneficial with view to inhibitory degradation products in the filtrates. Formic acid concentrations are much lower and fewer contaminations with phenolic compounds were obtained as degradation products from lignin. Besides of fewer inhibitors the MS filtrates have higher carbohydrate fractions. Indicating them to be more attractive compared to the CS filtrates. For further research, a comparison of MS and CS with CS silage is a topic of interest. CS silage might combine the benefits of both substrates investigated in this study, precisely a high accessibility of the substrate, the declaration as agricultural residue and the possibility of storage and full-year biomass supply.

## Data Availability

The original contributions presented in the study are included in the article, further inquiries can be directed to the corresponding author.
